# Association of Olfactory Impairment and Postoperative Cognitive Dysfunction in Elderly Patients

**DOI:** 10.3389/fmolb.2021.681463

**Published:** 2021-04-21

**Authors:** Yang Lan, Zhi-jian You, Ruiming Du, Le-si Chen, Jia-xuan Wu

**Affiliations:** ^1^Department of Anesthesiology, The Second Affiliated Hospital of Shantou University Medical College, Shantou, China; ^2^Department of Anesthesiology, Liuzhou People’s Hospital, Liuzhou, China

**Keywords:** postoperative cognitive dysfunction, IL-1β, TNF-α, anesthesia, olfactory function

## Abstract

**Objective:**

To investigate the impact of anesthesia on the change of olfactory function and cognitive function in elderly patients who undergo abdominal surgery.

**Methods:**

A total of 30 elderly patients who underwent abdominal surgery were recruited as the research subjects. The Connecticut Chemosensory Clinical Research Center (CCCRC) olfactory test was used to test the olfactory function and the Mini-mental State Examination (MMSE), Hopkins Verbal Learning Test – Revised (HVLT-R), Trail Making Test (TMT), Stroop Color Word Test (SCWT), Digit-Symbol Coding Test (DSCT), and Verbal Fluency Test (VFT) were used to assess their cognitive function before general anesthesia, and on the 3rd and 7th day post-anesthesia. The serum level of IL-1β, IL-6, and TNF-α were measured before anesthesia and at 0, 12, and 24 h post-anesthesia. In total, 30 healthy volunteers who did not undergo anesthesia were used as the control group. The test results of all subjects were recorded and their correlations were analyzed.

**Results:**

On the 3rd and 7th day post-anesthesia, the olfactory recognition threshold of patients in the surgical group was lower than that of control group with significant difference (*P* < 0.05). On the 3rd and 7th postoperative day, the patient’s short-term memory and delayed memory, attention and processing speed were decreased (*P* < 0.05). On the 7th day post-anesthesia, delayed memory and processing ability were still decreased (*P* < 0.05). In the surgical group, Spearman correlation analysis showed that the difference of olfactory recognition score on the 3rd and 7th day post-anesthesia was positively correlated with short-term memory and delayed memory of cognitive function. Compared with pre-anesthesia, the serum levels of IL-1β, IL-6, and TNF-α in the surgical group were significantly increased at each time point after anesthesia.

**Conclusion:**

Abdominal surgery with general anesthesia in elderly patients may increase the level of serum inflammatory factors, induce olfactory impairment, particularly the decline of olfactory identification threshold and cause cognitive dysfunction with declined short-term memory, delayed memory and attention. There was a positive correlation between olfactory impairment and cognitive dysfunction after general anesthesia. Therefore, olfactory impairment could be an early indicator to guide early intervention for postoperative cognitive dysfunction.

## Introduction

Postoperative cognitive dysfunction (POCD) refers to a complication of the central nervous system that occurs after surgery. Clinically, it manifests as decreased memory, attention, language understanding ability and change of personality. The occurrence of POCD can lead to slow recovery of patients, prolonged hospitalization, increased mortality and increased medical costs. Therefore, early detection and early intervention have a positive impact on the prognosis of POCD. Currently, there is no predictive indicator of POCD.

In as early as 1975, Ansari et al., found in their study of Parkinson’s disease (PD) that the olfactory threshold of 22 male PD patients was significantly higher than that of the healthy controls. They suggested that PD patients had olfactory dysfunction. Further studies proved that olfactory dysfunction was associated with other neurodegenerative diseases (Ansari and Johnson, 1975; Ansari, 1976). In the development of neurodegenerative diseases, olfactory dysfunction is an early manifestation of the disease (Arnold, 2007; Ottaviano et al., 2015). Nico et al., indicated that the mechanism of POCD was similar to that of neurodegenerative diseases (Bohnen et al., 2010; Ottaviano et al., 2015; Doty, 2017).

The causes of POCD are multifactorial, generally associated with patients’ age, anesthesia, intraoperative pathophysiological changes, surgical trauma, inflammatory response, hypoxia, thrombosis, and so on. Trauma, surgery, and anesthesia can stimulate the expression of cytokines such as IL-1β, IL-6, and TNF-α. These cytokines regulate many physiological activities of the central nervous system, and are involved in the pathophysiological process of central nervous system diseases.

In this study, we investigate the correlation between olfactory function and cognitive function in elderly patients who underwent general anesthesia, and evaluate whether the change of olfactory function is associated with the development of POCD.

## Materials and Methods

### Study Subjects

This study was approved by the Medical Ethics Committee of the Second Affiliated Hospital of Shantou University Medical College. A total of 30 elderly patients who underwent abdominal surgery at the ASA I-III level were designed as the surgical group. The 30 healthy volunteers whose gender, age and weight were matched with the patient group were designated as the control group. All participated individuals signed the informed consents. The subjects with any of the following conditions were excluded from the study: (1) Nasal or nasal sinus disease; (2) Obstructive lung disease; (3) Within 3 weeks after a cold; (4) Individuals with neuropsychiatric diseases such as Alzheimer’s disease (AD), PD, multiple sclerosis, or schizophrenia; (5) Individuals who are taking antipsychotics, antidepressants, or other medications that affect the central nervous system; (6) Individuals who underwent neuropsychological testing; (7) Individuals who are unwilling to comply with testing procedures; and (8) Alcoholics or drug addicts.

### Anesthesia

Anesthesia were carried out by using the inducing drugs of Midazolam 0.03∼0.05 mg/kg, Sufentanil 0.3–0.5 μg/kg, Rocuronium Bromide 0.6 mg/kg and Propofol 1.5–2.0 mg/kg, and then tracheal intubation. Continuous intravenous infusion of propofol 4–12 mg⋅kg^–1^⋅min^–1^, intermittent intravenous injection of Rocuronium Bromide 0.5 mg/kg, continuous intravenous injection of Remifentanil 0.05∼0.20 μg⋅kg^–1^⋅min^–1^ were used to maintain the anesthesia. The patients were put in volume control ventilation with the tidal volume set at 6–8 ml/kg. The time ratio of inspiration-exhalation was 1:2. The ventilation frequency was adjusted and maintained at PETCO2 35∼45 mmHg (1 mmHg = 0.133 kPa). After surgery, the patient was admitted to the PACU. The endotracheal tube was retained until spontaneous breathing resumes.

### Assessment of Olfactory Function

All subjects were tested with the Connecticut Chemosensory Clinical Research Center (CCCRC) olfactory assessment. The assessment has olfactory detection threshold (ODT) test and olfactory identification threshold (OIT) test. Olfactory detection threshold test uses the n-butyl alcohol and bromide, starting from a low concentration according to a ratio of 1: 2 continue to the highest concentration of 4%. The deionized water is used as the control. The value of threshold was set at which n-butanol can be correctly identified at the same dilution for four consecutive times. If one of the four times cannot be correctly identified, the concentration is increased until four times can be recognized continuously, and both sides of the nostril should be tested within 20 min. The olfactory identification test uses baby powder, star anise, sesame oil, mothballs, sulfur soap, pepper, and ammonia as the olfactory substances. These substances were, respectively, put into individual opaque plastic bottle, covered with gauze to avoid visual cues. Both sides of the nostrils were tested separately for 15 min. The scores of olfactory detection threshold are based on Table 1.

**TABLE 1:** CCCRC test series dilution and ODT scoring scale.

**Sequence**	**1**	**2**	**3**	**4**	**5**	**6**	**7**	**8**	**9**	**10**	**11**	**12**
Concentration (%)	4	1.3	0.44	0.15	0.049	0.016	5.5 × 10^–3^	1.8 × 10^–3^	6.1 × 10^–4^	2 × 10^–4^	6.8 × 10^–5^	2.3 × 10^–5^
Score	0	1	2	3	4	5	6	7	8	/	/	/

On a list of eight smells (along with charred paper ash, cinnamon, tobacco, peanut butter, ketchup, coffee, rubber, and wood shavings), the subjects were asked to choose which one they smelled, scoring one point for being right, and no points for being wrong or not knowing.

### Neuropsychiatric Test

The MMSE, SAS, SDS, ADL, HVLT⋅R, TMT, SCWT, DSCT, and VFT were used to evaluate the cognitive functions of patients. The mean test score (M) and the standard deviation (σ) were obtained. The difference of test score (ΔM) before and after anesthesia was used to calculate the Z score using the following formula:

$$Z=\frac{\Delta \,M}{\sigma }$$

*Z*-score ≥ 1.96 indicated POCD.

All the tests were performed by the same skilled operator and the basic information of the subjects, including gender, age, height, weight, ethnicity, marriage, education, ASA classification, current diagnosis, disease, allergy, heart rate, pulse oxygen saturation, operation time, bleeding volume, infusion volume, and blood pressure (mean arterial pressure) and VAS score were recorded.

### Testing the Inflammatory Factors

Before anesthesia and 0, 12, and 24 h post-anesthesia, 3 ml of venous blood was collected and the blood samples were centrifuged at 3,000 rpm for 15 min and then stored at −80°C until used. The level of serum inflammatory factors was examined using the commercially available ELISA detection kits: IL-1β (EK0392, Wuhan Boster Biological Technology., Ltd., Wuhan, China), IL-6 (EK0410, Wuhan Boster Biological Technology, Ltd., Wuhan, China) and TNF-α (EK0525, Wuhan Boster Biological Technology, Ltd., Wuhan, China).

### Statistical Analysis

Unless specified otherwise, the results were expressed as mean ± SD. Descriptive analysis was applied to assess the accuracy of values of assessment of cognitive function. The values were compared over three time points. Factorial ANOVA of 2 (group: control, surgery) × 3 (phase: pre-, 3rd day, 7th day) design was undertaken in SPSS 20.0, with repeated second factor (phase) measures. The data of two groups were compared via Chi-square test. The correlation between the data in two groups were compared via Spearman correlation coefficients. Significance was set at *p* < 0.05.

## Results

### Demographic Characteristics

This study included 30 cases of elderly patients who underwent abdominal surgery. There were 13 males and 17 females, aged 65–78 years old, years of education 0–9 years. Healthy control group consisted of 30 individuals, 15 males and 15 females, aged 65–80 years old, education level 0–15 years. There was no significant difference in gender, age and educational level between these two groups (*P* > 0.05). The demographics of participants by group (control vs. surgery) including age, gender, the level of education, hypertension and diabetes were summarized in Table 2.

**TABLE 2:** Demographic characteristics of groups (surgery and control) ($\bar{\text{x}}$ ± s).

	**Surgery**	**Control**
Number	30	30
Age (year)	64.8 ± 14.2	66.2 ± 11.8
M:F	13:17	15:15
Body Weight	68.1 ± 9.2	70.3 ± 8.5
ASA (number, I/II/III)	10/15/5	11/13/6
Education (year)	5.2 ± 3.7	5.5 ± 4.0
Diabetes (%)	30	33.3
Hypertension (%)	20	16.7
Smoking history (%)	33.3	36.7

### Comparison of Olfactory Function Between the Two Groups of Subjects

**FIGURE 1:**
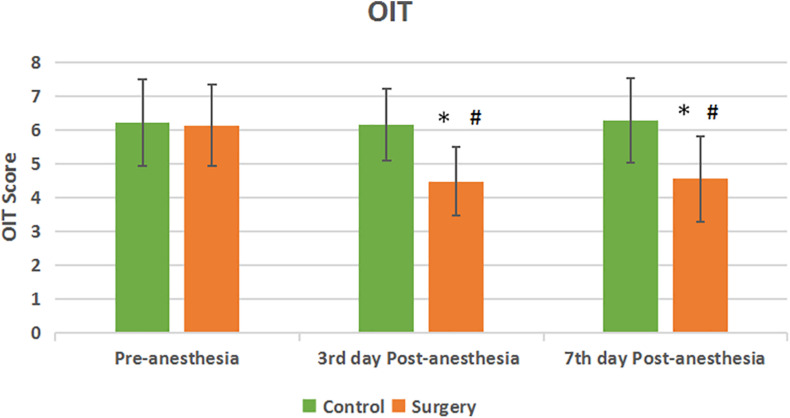
Olfactory identification threshold between the two groups. Compared with Pre-anesthesia, the OIT score decreased at the 3rd and 7th day Post-anesthesia in the patients of surgical group. The difference between the two groups was statistically significant. Values are mean ± SEM. *N* = 30, *p < 0.05 vs. the control group; #*p* < 0.05 vs. pre-anesthesia.

**FIGURE 2:**
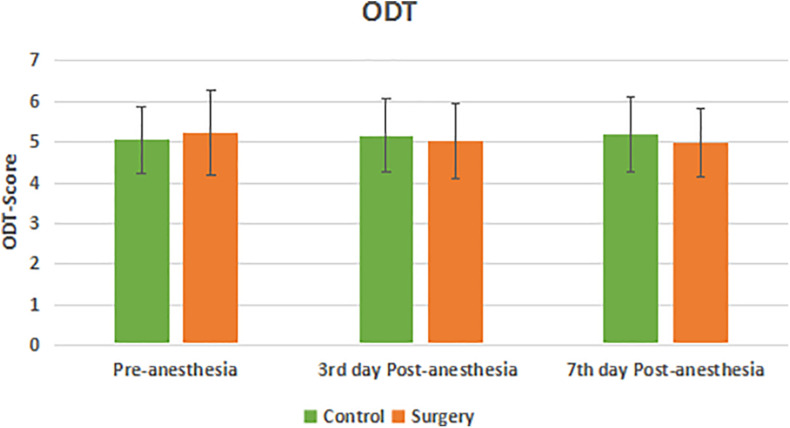
Olfaction detection threshold between the two groups. There was no significant decrease in ODT score at 3rd and 7th day Post-anesthesia in the surgical group compared with pre-anesthesia. There was no significant difference in ODT score in the surgical group compared with the control group.

There was no statistical difference in the olfactory function of subjects in the surgical and control groups before surgery. Compared with the control group, the olfactory identification threshold (OIT) in the patients of surgical group decreased on the 3rd and 7th day after anesthesia with statistical significance (*P* < 0.05) (Figure 1). There was no statistical significance in the olfaction detection threshold (ODT) between the two groups (*P* > 0.05) (Figure 2).

### Comparison of Cognitive Function Between the Two Groups

There was no significant difference in the preoperative cognitive function of subjects between the surgical group and the control group. Compared with the control group, the MMSE, short-term memory, delayed memory, attention and processing ability decreased in the patients of surgical group on the 3rd day after anesthesia (Table 3). The delayed memory and processing ability continue to decrease on the 7th day after anesthesia. The difference was statistically significant (*P* < 0.05).

**TABLE 3:** Comparison of cognitive function between the two groups.

**Projects**		**Group**	**Pre-anesthesia**	**3rd day post-anesthesia**	**7th day post-anesthesia**
MMSE		Control	24.3 ± 2.5	25.8 ± 3.6	25.1 ± 3.4
		Surgery	25.3 ± 3.8	18.9 ± 2.2	26.5 ± 3.1
		*P*-value	0.872	0.000	0.451
HVLT-R	Short-term retention	Control	21.2 ± 4.5	20.5 ± 5.1	21.9 ± 3.1
		Surgery	20.2 ± 4.4	17.5 ± 1.9	16.5 ± 3.0
		*P*-value	0.773	0.002	0.009
	Delayed retention	Control	7.5 ± 2.3	7.3 ± 4.2	7.3 ± 3.4
		Surgery	7.6 ± 2.5	5.2 ± 2.2	5.3 ± 1.8
		*P*-value	0.721	0.014	0.000
TMT		Control	42.4 ± 8.6	44.9 ± 9.4	43.1 ± 8.5
		Surgery	43.7 ± 8.4	52.1 ± 7.3	50.5 ± 9.9
		*P*-value	0.848	0.000	0.029
SCWT		Control	12.5 ± 2.3	11.5 ± 2.2	12.4 ± 2.6
		Surgery	11.8 ± 2.9	16.8 ± 2.6	11.4 ± 2.2
		*P*-value	0.847	0.001	0.239
DSCT		Control	45.1 ± 9.2	46.2 ± 9.9	46.4 ± 10.3
		Surgery	46.5 ± 9.1	47.5 ± 10.3	46.4 ± 11.0
		*P*-value	0.845	0.934	0.459
VFT		Control	14.5 ± 1.8	14.3 ± 1.4	15.1 ± 2.8
		Surgery	14.8 ± 1.9	15.2 ± 2.7	14.5 ± 2.4
		*P*-value	0.837	0.317	0.981

### Comparison of *Z*-Score at Different Time Points Between the Control and Anesthetic Groups

**FIGURE 3:**
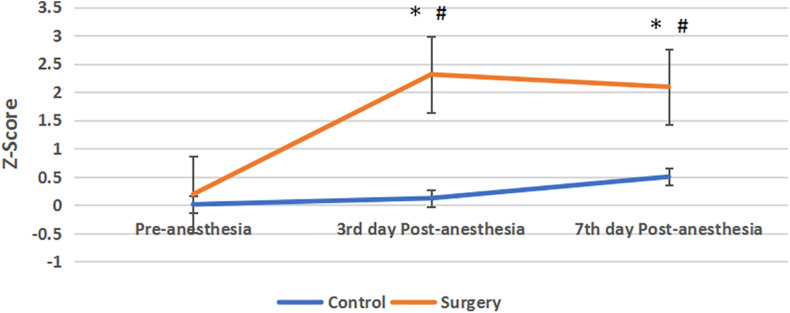
*Z*-score between the two groups. Compared with pre-anesthesia, the *Z*-scores of patients in the surgical group were significantly increased at the 3rd and 7th day Post-anesthesia, *Z*-score > 1.96 indicate the cognitive function was impaired. Compared with the control group, the *Z*-scores in the surgical group were increased and their cognitive function was impaired, and the difference between the two groups was statistically significant. Values are mean ± SEM. *N* = 30,*p < 0.05 vs. the control group; #*p* < 0.05 vs. pre-anesthesia.

Compared with the control group, *Z*-score was no statistical significance in the surgical group before anesthesia. The *Z*-score of the surgical group was higher than that of the control group on the 3rd and 7th day after anesthesia, and the difference was statistically significant. On the 3rd day after anesthesia, 12 patients had a *Z*-score ≧ 1.96, and 9 of them still had a Z-score ≧ 1.96 on the 7th days after surgery. In the control group, no subjects had a *Z*-value ≧ 1.96 at these two time points (Figure 3).

### Comparison of the Incidence of POCD Between the Control and Anesthetic Groups

According to the Z-score, the number of patients with POCD in both groups was obtained. In the control group, none of the subjects developed POCD. In the surgical group, POCD occurred in 7 patients, accounting for 23.33% of the surgical patients. The incidence of POCD between the two groups was statistically significant (Table 4).

**TABLE 4:** The incidence of POCD between the two groups (n).

	**Non-POCD**	**POCD**	**Incidence percentage (%)**
Surgery	23	7	23.33
Control	30	0	0
*P*-value			0.002

### Spearman Correlation Analysis of Olfactory Function and Cognitive Function in the Surgical Group

The olfactory detection threshold in patients in the surgical group was not significantly correlated with the MMSE, short-term memory, delayed memory, attention and processing ability. The difference of olfactory identification was positively correlated with the short-term memory (*r* = −0.455) and delayed memory (*r* = −0.348) 3 days after anesthesia, but not with the MMSE, attention and processing ability (Table 5).

**TABLE 5:** Spearman correlation analysis of olfactory function and cognitive function in the surgical group (*r-*value).

	**3rd day post-anesthesia**		**7th day post-anesthesia**	
	**ODT**	**OIT**	**ODT**	**OIT**
MMSE	–0.131	–0.220	–0.140	–0.122
*P*-value	0.605	0.162	0.774	0.592
Short-term retention	–0.156	–0.455	–0.138	–0.255
*P*-value	0.567	0.000	0.904	0.331
Delayed retention	–0.288	–0.348	–0.208	–0.232
*P*-value	0.431	0.012	0.434	0.586
SCWT	0.160	0.116	0.086	0.074
*P*-value	0.694	0.322	0.770	0.705
TMT	0.152	0.132	0.134	0.125
*P*-value	0.882	0.526	0.183	0.442

### Comparison of Serum Inflammatory Factors Between the Control and Anesthetic Groups at Different Time Points

Compared with pre-anesthesia, the level of IL-1β and IL-6 in serum of patients in the surgical group increased, and the level was highest at 0 h post-anesthesia. Although the level of inflammatory factors decreased at 12 and 24 h post-anesthesia compared with that at 0 h, it was significantly higher than that of pre-anesthesia, with statistical significance (*P* < 0.05). Among the 30 patients in surgical group, 7 patients with olfactory dysfunction showed significantly higher levels of IL-1β and IL-6 in serum at each time point after surgery than before surgery. At 12 and 24 h, the level of IL-1β and IL-6 decreased slightly compared with that at the end of surgery, but it was significantly higher than that before surgery, with statistical significance (*P* < 0.05). Compared with pre-anesthesia, the serum level of IL-1β, IL-6, and TNF-α of those 7 patients with olfaction dysfunction in the surgical group was significantly increased at each time point post-anesthesia (Table 6).

**TABLE 6:** Comparison of serum inflammatory factors between the two groups.

**Inflammatory factor**	**Group**	**Pre-anesthesia**	**0 h post-anesthesia**	**12 h post-anesthesia**	**24 h post-anesthesia**
IL-1β	Control	22.6 ± 3.5	23.1 ± 2.8	23.6 ± 3.1	22.9 ± 2.4
	Surgery	21.7 ± 2.8	38.6 ± 3.2	38.5 ± 3.3	30.6 ± 2.5
	*P*-value	0.618	0.001	0.021	0.000
IL-6	Control	19.5 ± 2.6	21.1 ± 3.1	20.6 ± 1.8	20.8 ± 2.5
	Surgery	20.6 ± 1.4	32.1 ± 2.4	28.1 ± 2.7	28.7 ± 3.1
	*P*-value	0.343	0.016	0.009	0.002
TNF-α	Control	35.6 ± 3.4	34.8 ± 3.1	34.9 ± 2.8	35.1 ± 2.7
	Surgery	36.3 ± 2.5	36.8 ± 2.8	43.4 ± 2.9	41.4 ± 3.2
	*P*-value	0.665	0.003	0.000	0.000

## Discussion

Postoperative cognitive dysfunction is a neurodegenerative disease after surgery and anesthesia, which is similar to AD and PD with symptoms and risk factors (age, education level, etc.) (Berger et al., 2015). The pathogenesis of POCD and AD, such as microtubule decomposition, deposition of tau protein and amyloid β (Aβ), are similar. It was suggested that POCD is a postoperative neurodegenerative disease (Wu et al., 2018). Decrease of amygdala neurons has been shown in PD patients with olfactory disturbance. Studies have shown that large amounts of neuronal tangles, senile plaque formation and neuronal necrosis can be found in the hippocampus and amygdala of AD patients (Reinsfelt et al., 2013; Lupton et al., 2016; Khan et al., 2017). The hippocampus and amygdala are associated with olfactory function, where information processing and processing of smell are completed. Dysregulation of hippocampus and amygdala might lead to olfactory disorders in both AD and PD patients (Kubota et al., 2020; Ubeda-Bañon et al., 2020). In the previous studies on AD patients with olfactory impairment, it was found that the level of serum acetylcholine was significantly reduced, which not only affects memory function, but also plays an important role in the olfactory system, indicating that the reduction of acetylcholine may also be a cause of the olfactory impairment in AD patients (Beach et al., 2000).

Currently, there is no clear diagnostic criteria and biomarkers for diagnosis of POCD (Silbert et al., 2011; Hussain et al., 2014). The diagnosis of POCD relies on a variety of psycho-mental test scales, and tests are conducted before and after surgery to obtain the dynamic change of patients’ cognitive function (Daiello et al., 2019). If some simple and accurate tests can be carried out on patients at an earlier stage, it is beneficial to intervene the occurrence and development of POCD as soon as possible. In this study, we used CCCRC method to test the olfactory function from two aspects: the olfactory detection threshold and olfactory identification threshold. The assessment of cognitive function included the language ability, memory ability, mathematical processing ability and attention. In this study, six scales including the MMSE, HVLT⋅R, TMT, SCWT, DSCT, and VFT were used to cover all aspects of cognitive functions. Subjects in the two groups were similar in age, sex and education. Postoperative analgesia can minimize pain and allow patients to better undergo tests of olfactory and cognitive function. According to the test results, the correlation between them is discussed.

In a study on PD patients, it was found that although most PD patients had damage on the olfactory identification threshold, their reactions to some strong odors indicated that the damage to olfactory function of PD patients mainly occurred in the cognitive processing of odor identification, rather than the ability to detect different odors (Kranick and Duda, 2008). This study also showed that the olfactory identification threshold and olfactory detection threshold of PD patients were both higher than those of non-PD patients, and the olfactory identification threshold was significantly higher than that of non-PD patients, indicating that the degree of damage was more serious than that of olfactory detection threshold (Hudry et al., 2003; Tremblay et al., 2020). The results of this study showed that 13 of the 30 elderly patients with abdominal surgery had olfactory impairment on the 3rd day post-anesthesia, with an incidence of 43.3%. Among them, 11 cases had olfactory identification impairment, seven cases had olfactory detection impairment. POCD occurred in 5 patients in the surgical group, with an incidence of 16.7%. Four patients showed impairment of one cognitive function index but did not meet the diagnostic criteria of POCD. Among these four patients, olfactory impairment occurred in two cases, accounting for 50%.

Inflammation is a pathological defense response to injuries and the most important protective response. Surgery can activate the immune system of the body and produce a strong inflammatory response. Peripheral inflammatory factors can directly or indirectly cause inflammatory response in the central nervous system (CNS) and affect cognitive function (Skvarc et al., 2018). The expression of inflammatory cytokines IL-1β, IL-6, and TNF-α in the hippocampus of rats undergoing partial hepatectomy has been shown to enlarge and prolong the cytokine response, especially the neuroinflammatory response, leading to changes in postoperative cognitive function. In this study, it was found that IL-1β and IL-6 in the surgical group increased after surgery compared with pre-anesthesia, while there was no significant change in the control group at the same time point. The expression of TNF-α has not increased significantly at the end of anesthesia compared with pre-anesthesia, there was no significant difference between the two groups, which may be related to the evaluation method, diagnostic criteria, or the number of the sample. In the case of small sample size, sensitive indicators are easier to be measured.

The occurrence of POCD in elderly patients undergoing abdominal surgery with general anesthesia is accompanied by the impairment of olfactory function, particularly the impairment of olfactory identification, and the olfactory impairment is correlated with development of POCD.

## Conclusion

The olfactory function, especially the olfactory identification threshold is damaged in elderly patients with general anesthesia. The degree of decreased olfactory function is positively correlated with the development of POCD. Therefore, evaluating the olfactory perception of postoperative patients could predict the declining of cognitive function and guide early intervention for postoperative cognitive dysfunction.

## Data Availability Statement

The original contributions presented in the study are included in the article/supplementary material, further inquiries can be directed to the corresponding author/s.

## Ethics Statement

The studies involving human participants were reviewed and approved by the Medical Ethics Committee of The Second Affiliated Hospital of Shantou University Medical College. The patients/participants provided their written informed consent to participate in this study.

## Author Contributions

JW conceived and designed the study, and revised the manuscript. YL, RD, and Z-JY collected and analyzed the case data. YL wrote the first draft. L-SC collected blood samples and detect inflammatory factors. All authors read and approved the final draft.

## Conflict of Interest

The authors declare that the research was conducted in the absence of any commercial or financial relationships that could be construed as a potential conflict of interest.
